# Changes in the nutritional status of elderly patients after esophagectomy

**DOI:** 10.1007/s10388-019-00681-0

**Published:** 2019-06-26

**Authors:** Kenjiro Ishii, Yasuhiro Tsubosa, Masahiro Niihara, Toshiya Akai, Wataru Soneda

**Affiliations:** Department of Esophageal Surgery, Shizuoka Cancer Center, 1007 Shimonagakubo, Nagaizumi-cho, Suntou-Gun, Shizuoka 411-8777 Japan

**Keywords:** Esophagectomy, Body mass index, Prealbumin, Nutritional status

## Abstract

**Background:**

Esophagectomy is a highly invasive procedure for patients aged > 70 years. Here, we compared the actual nutritional status of older and younger patients who underwent esophagectomy.

**Methods:**

A total of 118 patients who underwent radical esophagectomy between April 2013 and December 2016 were enrolled and divided into two groups based on age: group A (*n* = 41; ≥ 70 years) and group B (*n* = 77; < 70 years). Data pertaining to body mass index and nutritional variables (albumin; total cholesterol; cholinesterase; and prealbumin) were retrospectively analyzed preoperatively and at 3, 6, and 12 months postoperatively.

**Results:**

Significant preoperative between-group differences were found in the cholinesterase, albumin, and prealbumin levels. The body mass index gradually decreased over the first 12 months after surgery in both groups, without significant between-group differences. Significant differences were observed in prealbumin and cholinesterase levels at 3 months postoperatively. 1 year postoperatively, both groups showed slight improvements; however, the between-group differences were not statistically significant. Group A had a significantly lower amount of the degree of decrement of BMI and chE than group B.

**Conclusion:**

Thus, patients aged > 70 years can recover within 12 months of esophagectomy.

## Introduction

Esophageal cancer predominantly affects elderly individuals, and its incidence peaks after 65 years of age. The number of elderly patients (age ≥ 70 years) undergoing esophagectomy has increased with the increasing global life expectancy. In a study involving 2315 patients who underwent esophagectomy, 30 and 5% were aged ≥ 70 and > 80 years, respectively [1].

Radical esophagectomy is a highly invasive procedure; it results in reduced dietary intake and poor nutritional status. It is associated with several complications occurring more frequently in patients aged > 70 years. After esophagectomy, feeding patients can be challenging. Previous studies have reported decreased nutritional intake and persistent weight loss, even over the long term, in patients who underwent esophagectomy [2, 3].

On average, patients who underwent esophagectomy have been reported to experience 10% loss in their body weight during the first year postoperatively [2], and the nutritional quality maintained by these patients is strongly linked to quality of life, response to future treatments, and survival [4–9]. Recently, surgical techniques and postoperative management have improved; hence, esophagectomy is becoming more acceptable for patients > 70 years. However, there is a lack of information on the long-term and specific nutritional status of late-elderly patients after esophagectomy. We compared the actual nutritional status of older (age ≥ 70 years) and younger (age < 70 years) patients after esophagectomy.

## Materials and methods

This retrospective study was conducted at the Shizuoka Cancer Center, Japan. The independent medical ethics committee of the institute approved this study and waived off the need for informed consent because of the study’s retrospective and observational design.

Between April 2013 and December 2016, we examined the medical records of 118 patients who underwent esophagectomy with gastric replacement for long-term nutritional assessment. Patients with recurrent disease were excluded, and some patients were lost to follow-up. After esophagectomy, surgeons performed reconstruction that resulted in a completely vertical, tubularized stomach via the retrosternal route with an anastomosis at the cervical location. A jejunostomy tube was inserted from the gastric tube through the jejunum in patients with gastric tube reconstructions. Nutritional support provided via tube feeding with a semi-digested nutrition agent commenced on postoperative day 1. The dose was gradually increased. After discharge, the patients received injections of a semi-digested nutrition agent (250 mL/300 kcal/day) until 3 months postoperatively.

The patients were divided into two groups according to age: group A (*n* = 41; age, ≥ 70 years) and group B (*n* = 77; age, <  70 years). Data pertaining to body mass index (BMI) and the levels of nutritional variables (albumin, ALB; total cholesterol, TC; cholinesterase, chE; and preALB) were retrospectively collected preoperatively and at 3, 6, and 12 months postoperatively. The extent of changes in BMI and preALB levels at 3, 6, and 12 months postoperatively was also assessed.

We used medians and 25th percentile, 75th percentile as descriptive statistics, and statistical significance was considered with a *p* value less than 0.05. Data for our univariate analysis were tested using the Pearson’s Chi-square test, Fisher’s exact test, Mann–Whitney *U* test, and repeated-measure analysis of variance as appropriate.

## Results

### Baseline characteristics

Group A comprised 30 men and 11 women (median age, 72 years), whereas group B comprised 65 men and 12 women (median age, 62 years). The median preoperative BMIs were 20.7 (19.2, 22.3) kg/m^2^ and 21.6 (19.8, 23.6) kg/m^2^ for groups A and B, respectively, and these were not significantly different at baseline. Although both groups showed several pathological disease stages, we found no significant between-group differences. In both groups, thoracoscopy was more frequently performed than thoracotomy; however, there was no significant difference between the two groups with respect to frequency. Regarding preoperative therapy, 24 patients (58.5%) had chemo (radio)therapy in group A and 57 patients (74.0%) had the same in group B; however, there was no significant difference (Table 1, Fisher’s exact test and Pearson’s Chi-square test, Mann–Whitney *U* test).

**Table 1:** Patient characteristics

	Group A (*n* = 41)	Group B (*n* = 77)	*p* value
Male/female ratio	30/11	65/12	0.14
Age	74 (72, 76)	62 (57, 66)	< 0.05
BMI (kg/m^2^) (normal range 18.5–25)	20.7 (19.2, 22.3)	21.6 (19.8, 23.6)	0.88
Tumor staging (UICC 7th)			0.10
pStage 0	0	0	
pStage I A	18	20	
pStage I B	3	3	
pStage II A	6	7	
pStage II B	1	16	
pStage III A	6	7	
pStage III B	2	8	
pStage III C	2	8	
pStage IV	1	2	
pT0	2	6	
Type of surgical procedure			0.70
Open	18	31	
Thoracoscopic	23	46	
Kind of therapy before operation			0.08
Chemotherapy	23	51	
Chemoradiotherapy	1	6	

Descriptive statistics, median (25th percentile, 75th percentile)

Statistical method: Pearson’s Chi-square test, Fisher’s exact test, and Mann–Whitney *U* test

*BMI* body mass index, *UICC* Union for International Cancer Control

### Nutritional status

**Fig. 1:**
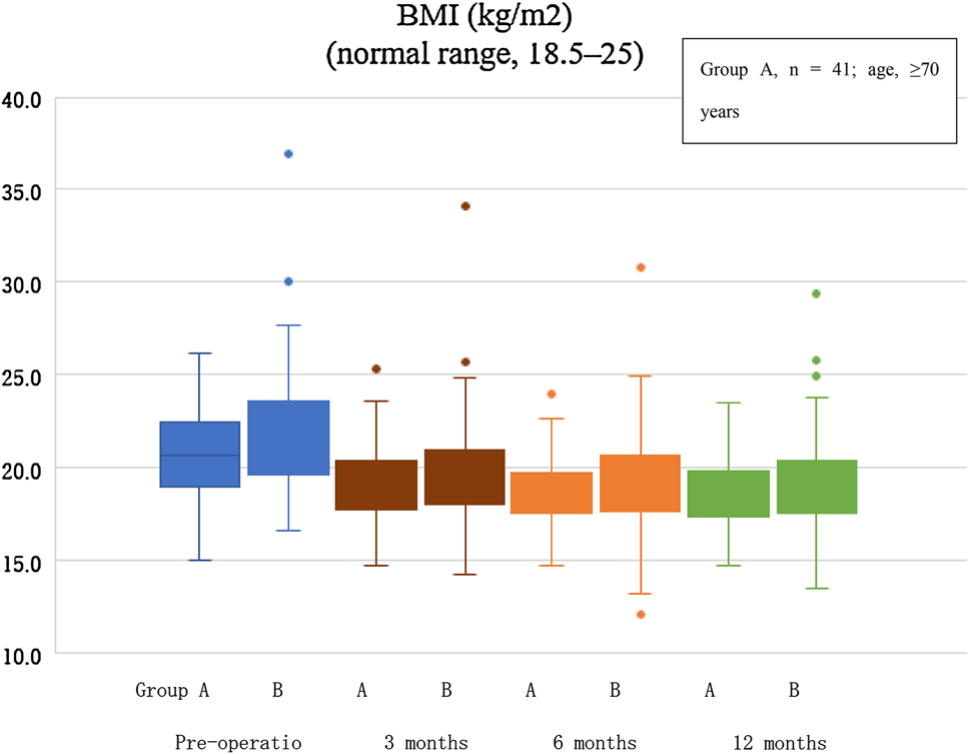
Change in body mass index. This figure shows the median BMI. There were no significant between-group differences at any time point. *BMI* body mass index

We found no significant between-group differences in the preoperative BMI levels. However, there were significant between-group differences in preoperative preALB and chE levels. BMIs declined gradually until 1 year postoperatively in both groups (group A, 19.0 (17.4, 19.8) kg/m^2^; group B, 18.9 (17.6, 20.4) kg/m^2^), without significant between-group differences. (Table 2, Fig. 1, Mann–Whitney *U* test).

**Table 2:** Preoperative and postoperative nutritional status of patients

	Group A (*n* = 41)	Group B (*n* = 77)	*p* value
BMI (kg/m^2^)			
Pre-operation	20.7 (19.2, 22.3)	21.6 (19.8, 23.6)	0.88
3 months	18.6 (17.7, 20.4)	19.2 (18.0, 21.0)	0.13
6 months	19.4 (17.6, 19.7)	19.2 (17.7, 20.6)	0.21
12 months	19.0 (17.4, 19.8)	18.9 (17.6, 20.4)	0.25
PreALB (mg/dL)			
Pre-operation	21.3 (19.1, 24.7)	26.3 (17.9, 23.6)	<0.05
3 months	18.2 (15.4, 20.9)	21.3 (19.1, 24.2)	<0.05
6 months	20.6 (18.3, 21.8)	21.5 (19.1, 24.2)	0.21
12 months	21.5 (18.2, 24.0)	22.9 (20.0, 25.3)	0.58
chE (IU/L)			
Pre-operation	257 (243, 352)	290.0 (243, 352)	<0.05
3 months	219 (214, 301)	241 (214, 300)	<0.05
6 months	236 (218, 289)	257 (218, 288)	0.26
12 months	235 (231, 310)	259.5 (231, 310)	0.11
TC (mg/dL)			
Pre-operation	201 (179, 223)	213.5 (186.5, 237.5)	0.19
3 months	186 (171, 206)	190.0 (173.0, 218.2)	0.19
6 months	192 (154, 214)	184 (168.0, 207.0)	0.92
12 months	185 (173, 224)	194.0 (173.5, 208.5)	0.62
ALB (g/dL)			
Pre-operation	4.1 (3.7, 4.2)	4.1 (3.8, 4.3)	0.18
3 months	4.1 (3.7, 4.3)	4.1 (3.8, 4.4)	0.34
6 months	4.1 (3.8, 4.3)	4.1 (4.0, 4.4)	0.26
12 months	4.1 (3.9, 4.3)	4.1 (4.0, 4.4)	0.77

Descriptive statistics, median (25th percentile, 75th percentile)

(Statistical method, Mann–Whitney *U* test)

*BMI* body mass index, *ALB* albumin, *ChE* cholinesterase, *TC* total cholesterol

**Fig. 2:**
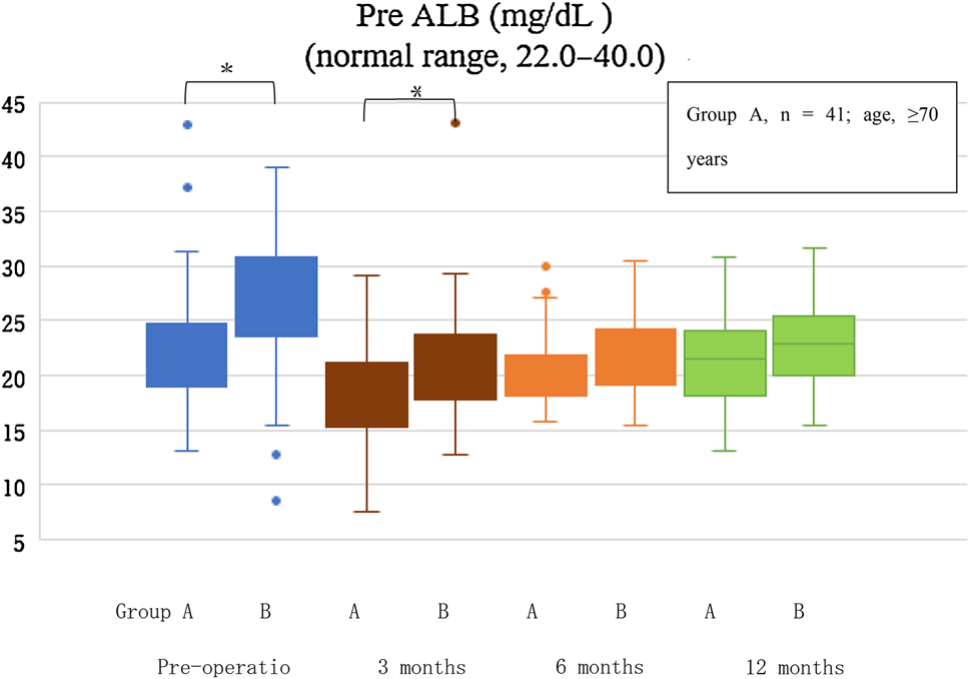
Change in prealbumin. This figure depicts the median values before and after surgery. There is a significant difference preoperatively and at 3 months postoperatively. *PreALB* prealbumin. *p value < 0.05

Regarding laboratory nutritional data, ALB and TC levels were almost normal pre- and postoperatively in both groups and no significant between-group differences were observed. There were significant between-group differences in preALB (group A, 18.2 (15.4, 20.9) mg/dL; group B, 21.3 (19.1, 24.2) mg/dL; *p* < 0.05) and chE (group A, 219 (214, 301) IU/L; group B, 241 (214, 300) IU/L; *p* < 0.05) levels at 3 months postoperatively. However, the values gradually improved in both groups, reaching normal levels by 1 year postoperatively. Furthermore, there were no significant between-group differences in preALB and chE levels at 6 and 12 months postoperatively (Table 2; Fig. 2, Mann–Whitney *U* test). In regard to the degree of decrement of BMI, preALB, and chE, group A had a significantly lower amount of decrement of BMI and chE than group B (BMI *df* = 1, *F* = 289, *p* < 0.05; chE *df* = 1, *F* = 35.6, *p* < 0.05), and there was no significant difference in the decrement of preALB between both groups (*df* = 1, *F* = 15.4, *p* = 0.06) (Table 3, repeated-measure analysis of variance).

**Table 3:** Decrement of BMI, preALB, and chE

	Group A (*n* = 41)	Group B (*n* = 77)	*p* value
BMI (kg/m^2^)			<0.05
3 months	1.7 (0.8, 2.5)	2.3 (1.2, 3.1)	
6 months	2.0 (0.7, 2.9)	2.4 (0.9, 4.4)	
12 months	2.2 (0.5, 3.1)	2.4 (1.1, 4.3)	
PreALB (mg/dL)			0.06
3 months	5.7 (1.3, 8.3)	7.2 (3.1, 9.6)	
6 months	1.2 (− 1.3, 6.0)	5.1 (2.7, 8.5)	
12 months	0.8 (− 2.6, 4.4)	3.8 (1.1, 7.5)	
chE (IU/L)			<0.05
3 months	18.5 (0.0, 51.0)	46.0 (1.8, 69.8)	
6 months	4.0 (− 15.5, 28.5)	33.0 (45.0, 78.0)	
12 months	6.0 (− 25.5, 36.3)	29 (1.0, 70.0)	

Statistical method, repeated-measure analysis of variance

*BMI* body mass index, *ALB* albumin, *ChE* cholinesterase

## Discussion

The global life expectancy is increasing; hence, we anticipate that by 2050 at least 16% of the world’s population will be aged ≥ 65 years [10]. According to the American Geriatrics Society, individuals aged ≥ 75 years are considered “elderly” [11]. In Japan, people aged ≥ 70 years constituted 18.7% of the entire population in 2016. Studies have reported on the rates of postoperative complications, hospital stay duration, short-term morbidity and mortality, and 5-year overall and disease-specific survival in elderly patients [12–15]. Wright et al. reported that compared with patients aged 55–74 years, those aged ≥ 75 years display worse outcomes [1]. Although esophagectomy can be justified in patients aged 70–79 years owing to low mortality in this age group, Luis et al. reported a significant increase in the major complications after esophagectomy in patients > 80 years [16].

On average, patients who underwent esophagectomy have been reported to experience 10% loss in their body weight during the first year postoperatively [2, 4]. Nutrition following upper gastrointestinal surgery is related to postoperative treatment, survival rate, and quality of life [5, 8, 17, 18]. In contrast, a study including some nutritional scores has reported nutritional status until 6 months postoperatively [19].

Here, we calculated the actual changes in the nutritional status of elderly patients. Patients’ BMIs decreased gradually until 1 year postoperatively, and preALB levels reached nadir at 3 months postoperatively; thus, the time points at which BMI and preALB values were at their minimum did not coincide. Total energy intake was the lowest at 3 months after a major upper gastrointestinal surgery [19]. preALB levels reflect recent dietary intake much more closely than the overall nutritional status [20], which explains the perceived gap.

There were no significant between-group differences in BMI preoperatively; however, preoperative preALB and chE levels were higher in group B than in group A. This might be so because there were more patients demonstrating slight nutritional excess in group B. The difference persisted for 3 months postoperatively, and the levels in both groups became similar at 6 and 12 months postoperatively. It is still possible that older patients are more affected by highly invasive operations, such as esophagectomy, in terms of food intake early after esophagectomy; however, the impact of those operations does not seem to be higher than that observed in younger patients over time.

chE levels were also nadir at 3 months postoperatively but remained at relatively normal levels at other times. TC and ALB levels were normal at all times and are, therefore, not suitable nutritional indices after esophagectomy.

BMI and preALB levels did not decrease as much as expected in patients aged > 70 years. This suggests that the semi-digestion nutrition agent on the enteral feeding tube, which was applied until 3 months postoperatively, led to a decline in the expected levels of reduction of these parameters.

1 year postoperatively, BMI and all nutritional variable levels were almost within normal ranges, even in patients aged > 70 years. Moreover, these patients had an even lower degree of decrement of BMI and chE than younger patients postoperatively. Of course, it is partly because younger patients had much higher BMI and chE levels preoperatively; however, this result indicates that even elder patients experience enough recovery after esophagectomy, within the first 12 months, which is beyond expectation.

There are some limitations to this study. First, this retrospective study was conducted at a single center and was limited to the Japanese population. Also, we have extensive experience and frequently perform esophagectomies due to the higher local incidence of esophageal cancer. Moreover, our outcomes may not be applicable to centers in other countries.

In conclusion, provided appropriate eligibility criteria are maintained, even patients aged > 70 years can overcome esophagectomy and regain significant strength within 12 months postoperatively.
